# Information seeking behavior of patients with diabetes mellitus: a cross-sectional study in an outpatient clinic of a university-affiliated hospital in Athens, Greece

**DOI:** 10.1186/s13104-015-1005-3

**Published:** 2015-02-20

**Authors:** Sofia Kalantzi, Petros Kostagiolas, Georgios Kechagias, Dimitrios Niakas, Konstantinos Makrilakis

**Affiliations:** First Department of Propaedeutic Medicine, Athens University Medical School, Laiko General Hospital, 17 Ag. Thoma St, 11527 Athens, Greece; Faculty of Social Sciences, Healthcare Services Management, Hellenic Open University, Patras, Greece

**Keywords:** Diabetes, Information behavior, Information needs, Sources, Obstacles

## Abstract

**Background:**

The purpose of this study is to examine the information behavior of diabetic patients, a relatively unexplored field of diabetes care, including their needs for information, resources used, obstacles encountered and degree of satisfaction for diabetes-related information acquisition.

**Methods:**

203 patients (males: 110, type 2:172) followed-up in the outpatient Diabetes Clinics of a University-affiliated hospital in Greece were assessed, using a validated questionnaire.

**Results:**

Patients identified diet (61.4%) and diabetic complications (41.9%) as “the most important” for their information needs and the treating physician (94.6%) for information resources. Internet importance and frequency of use ranked low. Main obstacles to information seeking were “lack of time” and “cost”. Most patients (71.4%) stated they were “quite” or “very satisfied” with the current possibilities of information seeking.

**Conclusions:**

Diabetic patients’ stated information needs and information sources, as well as main obstacles to obtaining information could potentially have important implications in designing a future information campaign.

## Background

Diabetes mellitus (DM) is a complex, chronic condition that requires both high quality clinical care and effective self-management to minimize its dire health and economic consequences [1-3]. Over the course of a lifetime, people will need a variety of skills and knowledge to enable them to control their condition, sometimes on a day-to-day basis, and modify their approach when circumstances change. The success of diabetes care relies mainly on patients’ daily self-care activities and providers’ continuous support. Diabetes, therefore, is a disease in which information and knowledge from the patients’ perspective has an important role to its management and as a result, diabetes self-management education (DSME) and on-going support are significant contributors to metabolic and psychological outcomes. The overall objectives of DSME are to support informed decision-making, self-care behaviors, problem-solving and active collaboration with the health care team and to improve clinical outcomes, health status, and quality of life [4]. Training and education to enable people to self-manage their diabetes helps prevent unnecessary health care utilization and hospitalizations [5]*,* and improves glycemic control [6].

“Information behavior” is the currently preferred term used to describe the many ways in which human beings interact with information; in particular, the ways in which people seek and utilize information [7]. A number of models have been proposed to characterize various aspects of information behavior [8,9]. These imply that the information is evaluated as to its effect on need, and forms part of a feedback loop that may start the process of seeking all over again if the need is not satisfied. Studies have identified the central role of information seeking and acquisition in enabling a person to cope with both the initial diagnosis and the ongoing impacts of a life-threatening illness [10]. However, it appears that relatively little has been investigated within the scientific community specifically focused on the information behavior of people with diabetes [11]. This is especially true in comparison to the much more extensive literature covering the information behavior of people with other diseases, such as cancer [12,13].

The purpose of the present study was to examine the information seeking behaviors of patients with Type 1 and Type 2 diabetes, including their stated needs for information, resources used, obstacles encountered and their degree of satisfaction regarding DM related information in a sample of diabetic patients attending a major University hospital, in Greece. To our knowledge, no such study has been performed in Greek patients so far.

## Methods

Participants were prospectively recruited from patients being followed up at the outpatient Diabetes Clinic and the outpatient Diabetic Foot Clinic of the University affiliated “Laiko” General Hospital, in Athens, Greece. All patients who presented to the outpatient Clinics during the month of February 2012 were asked to participate. Adult (>18 years old) Type 1 or 2 diabetic patients who were capable of understanding the questionnaire and give their written informed consent were eligible to be included. Exclusion criteria were lack of patients’ understanding oral Greek language or refusal to participate in this study. The study was approved by the participating Hospital’s Ethics Committee and carried out in accordance with the principles of the Declaration of Helsinki, as revised in 2008 [14].

Participants’ socio-demographic and diabetes specific data, including gender, age, urban or rural domicile, marital status*,* educational attainment, self-reported personal income, years since first diagnosis of DM, Type of DM (1 or 2), treatment (oral hypoglycemic agents (OHA), insulin or diet), number of visits to the outpatient diabetes clinic in the previous semester and most recently measured HbA_1c_ level (extracted from the patients’ medical charts) were recorded. Participants completed a brief printed, previously validated [11] questionnaire that consisted of 57 items and used plain language. Apart from demographic and DM related data, it assessed patients’ information needs (15 questions), information sources (18 questions), obstacles to information seeking (11 questions) and degree of satisfaction for their current ability to seek information (1 question) (Tables 1,2,3 and 4). The patients completed the questionnaire independently, except in those cases that a disability, such as poor vision due, for example, to DM retinopathy, or illiteracy precluded them from being able to dependably complete the questionnaire. In that case the questionnaire was read aloud in a neutral way by the investigator and the reported answers were annotated to the correspondent form.

**Table 1:** **Needs for information about diabetes**

**How important do you consider the following factors/knowledge urging you to seek information?**	**1 = not at all to 5 = most important**	**Do not know**
What is diabetes?		
What are the symptoms of diabetes?		
What are the causes of diabetes?		
What are the complications of diabetes?		
What is the proper diet for people with diabetes?		
What is the right exercise for diabetes?		
What are the new medications for diabetes and what are their unwanted effects?		
When should I start to take insulin?		
How should I use insulin?		
What are the measures taken to avoid foot complications of DM?		
What are the symptoms of hypoglycemia?		
What are the sexual problems due to diabetes?		
What should I do in case of another illness (such as infection)?		
I think I am sufficiently informed about diabetes/I do not seek information about diabetes.		
Other (please specify)

**Table 2:** **Sources of information used by diabetic patients**

**Which sources do you use to seek information about diabetes?**	**1 = not at all to 5 = constantly**	**Do not know**
Doctors		
Nurses		
Health visitors		
Eye doctor		
Dietitian		
Pharmacist		
Internet		
Books		
Leaflets		
Magazines/newspaper/newsmagazine articles		
Broadcast media (television, radio)		
Patients’ associations		
Family, including family members with diabetes/friends		
Other patients with diabetes		
Seminars for diabetics		
Booklets, brochures, etc., from clinic or health professionals		
Other

**Table 3:** **Obstacles to DM-related information seeking**

**How important are for you the following problems in seeking information about diabetes?**	**1 = not at all, to 5 = most important**	**Do not know**
Lack of time		
Cost		
Lack of competent infrastructures		
Lack of health care providers		
Lack of computer literacy		
Great volume of unorganized information		
Lack of understanding information due to scientific terms used		
Lack of understanding information written in a foreign language		
Psychological issues		
Problems in the doctor-patient relationship		
Other (please specify)

**Table 4:** **Degree of satisfaction with the current possibilities of acquiring information about DM**

Not at all (%)	0.49
A little (%)	1.97
Moderately (%)	26.1
Quite (%)	64.0
Very (%)	7.4

### Statistical analysis

Qualitative variables are presented as absolute and relative frequencies (%). Comparisons between categorical variables were tested with the use of contingency tables and the calculation of the Chi-square test and the Kendall’s tau test for ordinal data [15]. Associations and correlation coefficients between the clinical parameters were evaluated by the Spearman’s correlation test. All reported p-values are from two-sided tests and compared to a significance level of 5%. Data were analyzed using SPSS for Windows, version 19.0 (SPSS Inc. Chicago, Illinois).

## Results

Among the 604 eligible patients, 203 agreed to participate in the study and completed the questionnaire. Those who refused did so because of alleged problems with their vision, language problems or lack of time. Their age and gender was not different from those who accepted to participate. The demographic and diabetes-specific data of the participants are displayed in Table 5. No missing data were observed. They were fairly gender-balanced (male:female ratio 1.18:1) and consisted primarily of sextagenarian patients or older (57.6%), married (57.6%), that resided in an urban area (81.8%), having attained primary (48.3%) or secondary (36.0%) education and of a low personal income (<12,000.00 €, [69.5%]). Furthermore, the majority of them were suffering from type 2 DM (84.7%), with a long-established diagnosis (over 10 years, 48.8%), on OHA treatment (50.7%), having attended the outpatient diabetes clinic once or twice during the previous semester (60.1%) and most having a decent DM control (44.8% of them had HbA1C: 6.5-7.5%).

**Table 5:** **Demographic and diabetes-specific characteristics of participants (n = 203)**

**Characteristic**	**Value**
Sex, no. (%)	
Μale	110 (54.2)
Female	93 (45.8)
Age, no. (%)	
18-40 yrs	23 (11.3)
40-50 yrs	27 (13.3)
50-60 yrs	36 (17.7)
>60 yrs	117 (57.6)
Domicile, no. (%)	
Urban	166 (81.8)
Rural	37 (18.2)
Marital status, no. (%)	
Married	117 (57.6)
Divorced	24 (11.8)
Single	21 (10.3)
Widowed	41 (20.2)
Education attainment (level), no. (%)	
None	3 (1.5)
Primary	98 (48.3)
Secondary	73 (36.0)
Tertiary	29 (14.3)
Estimated annual personal income, no. (%)	
<12,000 €	141 (69.5)
12,000-18,000 €	47 (23.2)
18,000-25,000 €	12 (5.9)
>25,000 €	3 (1.5)
Type of clinic attended by patient, no. (%)	
Outpatient diabetes clinic	123 (60.6)
Outpatient diabetic foot clinic	80 (39.4)
Type of diabetes, no. (%)	
Type 1	29 (14.3)
Type 2	172 (84.7)
Unsure	2 (1.0)
Time since diagnosis, no. (%)	
<1 years	8 (3.9)
1–5 years	37 (18.2)
5-10 years	59 (29.1)
>10 years	99 (48.8)
Treatment of diabetes, no. (%)	
OHA	103 (50.7)
Insulin	73 (36.0)
Diet alone	2 (1.0)
OHA and insulin	10 (4.9)
OHA and diet	8 (3.9)
Insulin and diet	2 (1.0)
OHA and insulin and diet	5 (2.5)
Number of visits during the last semester, nο (%)	
None	32 (15.8)
1-2	122 (60.1)
>3	49 (24.2)
Most recent HbA1c level, no. (%)	
<6.5%	36 (17.7)
6.5-7.5%	91 (44.8)
7.5-8.5%	40 (19.7)
>8.5%	16 (7.9)
Unknown	20 (9.9)

Participants were invited to answer how important they considered the items listed in Table 1 that motivated them to seek information on DM. Their answers indicated that the principal information considered as the most important by the majority of them (61.4%) concerned “what is the proper diet for diabetes”, which was associated with younger age (p = 0.037). The next most important item reported was “what are the complications of diabetes” (41.9% of participants), which was associated with younger age (p < 0.001), higher level of education (p = 0.002) and higher income (p = 0.002). Third in reported importance was “what is the right exercise for diabetes” (28.1% of participants), associated with younger age (p < 0.001), higher education (p < 0.001), higher income (p = 0.001), lower disease duration (p = 0.005), Type 1 DM (p < 0.001) and lower HbA1c levels (p = 0.010). Furthermore, fourth in reported importance was “the measures taken to avoid foot complications in DM” (24.1% of participants). This was increasingly important to patients who were being followed-up in the Diabetic Foot outpatient Clinic (p < 0.001) as well as to patients with a longer duration of DM (p = 0.003), those who resided in an urban area (p = 0.010), who had more visits to the clinic during the previous semester (p = 0.003) and who had a worse DM control (p = 0.014). The “symptoms of hypoglycemia” were ranked fifth in reported importance as a motivator to seek information for DM (21.7% of participants). People with Type 1 DM (p < 0.001), of younger age (p < 0.001), those followed in the Diabetes outpatient Clinic (p < 0.001), females (p = 0.011), with higher education (p < 0.001), shorter duration of the disease (p = 0.028) and a better DM control (p < 0.001) were more concerned about this variable. Interestingly, people with a longer duration of DM considered their knowledge and information about the disease quite satisfactory and reported they did not seek any more information about diabetes (p < 0.001). The same held true for participants with lower education and income, older age, females and those followed-up in the Diabetes Foot Clinic.

The diabetes-related sources of information that the study participants reported to use are summarized in Table 2. The majority of them (94.6%) reported they relied on their physicians as their main source of information (especially for patients with lower education, lower income and worse DM control), followed by the ophthalmologist (31.5% of participants, especially for males, with longer duration of DM, more visits to the clinic and better DM control). The broadcast media, i.e. television and radio, were ranked third in importance (15.3% of participants, especially for males, of younger age, higher education and better DM control). Relatives/friends (7.8%), books (6.4%), nurses (1.5%), pharmacists (1.0%), and other information sources were ranked much lower in importance. It is also of note that, although “internet usage” as a whole was limited (only 5.4% of the participants considered it as an important source of information for DM), in the younger age group of patients (<40 years), which was a minority of the patients that took part in this study (11.3%), the internet was considered as a quite important information source (30.4% vs. 2.2% for those >40 years old). Apart from age (p < 0.001), internet usage as an important information source was associated with having Type 1 DM, having higher education and income, lower duration of the disease, being followed-up in the Diabetes Clinic vs. the Diabetic Foot Clinic and having better DM control. Moreover, the questionnaire specifically explored the reported frequency of the internet usage as an information source for DM (Table 6). Most patients (71.9%) reported that they never used the internet, while in total 91.1% of the participants reported they used the internet at a frequency of once a month or less for seeking information about DM.

**Table 6:** **Reported frequency of internet usage as an information source for DM**

Every day, no. (%)	6 (3.0)
Once weekly, no. (%)	12 (5.9)
Once a month, no. (%)	26 (12.8)
Rarely in the year, no. (%)	13 (6.4)
Never, no. (%)	146 (71.9)

The obstacles to information seeking were investigated based on the questions listed in Table 3. The participants reported that important obstacles to information seeking were “lack of time” (33.5% of participants) and “cost” (31.5%), especially in younger patients <40 years old (78.3% and 65.2%, respectively), and those with higher education level and lower duration of the disease. Moreover, younger patients, with Type 1 DM and those with a higher education and higher income indicated that a large volume of unorganized information was a significant barrier to obtaining useful information. Patients residing in rural areas indicated more frequently that lack of competent infrastructures (78.4%) and lack of health care providers (81.1%) were obstacles to information, indicating a deficiency in structures and staff to sufficiently cover the information needs of diabetic patients in these areas. Psychological factors were more correlated to female gender.

Lastly, the participants were questioned about their degree of satisfaction for their current possibilities of acquiring information about DM (Table 4). The answers received indicate that 71.4% of the patients consider they are “quite” or “very” satisfied with the current possibilities of acquiring information. Patients with a higher level of education tended to report they were more satisfied (p = 0.049), which could be attributable to their broader use of information sources as they strive to fulfill their information needs. The same was true for patients being followed-up in the Diabetes Clinic as opposed to the Diabetic Foot Clinic (p = 0.023), patients with better DM control (p = 0.009) and those with longer duration of the disease (p = 0.008).

## Discussion

Provision of education and information forms a major part of chronic disease management strategies. People with chronic disease who receive education are presumed to be in a better position to take responsibility for their own health, participate in their own health-care and management, and thus maximize their health outcomes [16]. Diabetes mellitus is a prime example of a chronic disease that carries a significant socio-economic burden and has a prevalence that is expected to rise in the future [1]. DM is also a disease with the peculiarity that patient care is highly dependent on the patients themselves. Indeed, the diabetic patient often has to follow instructions and to adapt their diet and medications according to glucose measurements and to be on the alert for early symptoms of hypoglycemia, hyperglycemia or a variety of other complications (e.g. foot problems). A higher quality of self-care has indeed been shown to be beneficial to the diabetic patients, in terms of concrete clinical outcomes, such as HbA1c [4,6]. The diabetic patient is therefore called upon to constantly and successfully seek and manage information about their condition. Knowledge and information, however, is not necessarily translated into action or better health behaviors [17]. An understanding is required of the individual’s perspective of their disease, how they manage it, and how they think management, including education and provision of information, could be improved. Recent decades have witnessed a growing emphasis on patients as active consumers of health information in many fields of medicine [12]. The study of information behavior in DM is an emerging field, as indicated by the relative scarcity of the published studies on this subject [11,18]. To our knowledge, no such study has been performed in Greek patients so far.

In the present study, the diabetic patients’ information behavior was assessed, namely their perceived information needs, reported information sources and frequency of internet use as a source of relative information, reported obstacles to information and perceived degree of satisfaction of their current possibilities for information. According to their answers, “dietary issues” were considered as the most important diabetes-related matter to be informed about (61.4% of the participants), especially for younger patients. It is possible that the patients’ reported intense interest in diet reflects their understanding of the fundamental role of diet in DM management and self-care [19], but at the same time the complexity of the problem and the difficulties with adhering to a change in lifestyle for a long time. Patients placed second in importance for information the issue of “diabetic complications”, obviously reflecting their agony for the known dire consequences of the disease and their need to avoid or cope with them. Patients that were followed-up in the Diabetic Foot Clinic understandably seemed to be more interested in management of diabetic foot problems. These data are in agreement with the findings of the Australian North West Adelaide Health Study [18], where participants reported a need for more information on diet and the long-term effects of diabetes on eyes, kidneys, feet and the cardiovascular system.

Notably, diabetic patients with a long-established diagnosis of the disease reported they viewed their knowledge in DM as satisfactory and they did not actively seek information. That could indicate either a truly satisfactory level of information about DM in long term diabetic patients or, possibly, a misconception by the patients themselves that erroneously judge their level and quality of DM-related information as higher than it objectively is. Additionally, these patients stated their disinterest in seeking new information and this could translate into a relative unreceptiveness to an information campaign in a real life context. These data are in contrast to the findings of the study by Longo et al. [11], where many participants, even years after diabetes was diagnosed, expressed the need for periodic reeducation as they realized how much there is to learn, encountered confusing or conflicting information, or discovered that information changes over time. Differences in socioeconomic and cultural status between the participants of that and the current study could explain this disparity.

The data regarding the reported sources of information are very interesting: 94.6% and 31.5% declared their treating physician and the ophthalmologist, respectively, as an extremely important source of diabetes-related information, followed by the broadcast media such as television and radio (15.3%). On the other hand potential sources, such as nurses (1.5%) and the internet (5.4%) ranked quite low in the patient reported importance as an information source. These results come in partial contrast with information behavior studies in other western-culture populations. Despite the fact that the present study cannot be directly compared to the studies by Chittleborough et al. [18] and Longo et al. [11], it should be noted that in those studies the physicians were also recognized as an important resource of information, but the role of nurses was deemed significant as well. The differences in the status of nurses as a perceived diabetes information resource in the present study could reflect a doctor-centered approach and other intrinsic characteristics of the Greek National Health System. The concept of “diabetes educator” [20] virtually is lacking from the Greek Health Care system. That in turn could indicate a possible opportunity to empower and expand the role of nurses and other non-medical health care providers as an information resource for diabetic patients, potentially ameliorating health outcomes. Furthermore, internet ranks very low in stated importance, which is further validated by the low reported frequency of internet use as a tool for diabetes-related information by the great majority of participants. There is evidence that diabetic patients of younger age, higher level of attained education and income tend to use the internet more to this purpose, perhaps due to higher computer literacy, familiarity and accessibility to internet [21]. Similar results were reported by Longo et al. [11], where “despite the power of the Internet, patients reported they relied more on traditional sources of information, most particularly nurse practitioners, dietitians, and diabetes educators. Physicians, too, played a major role in diagnosis and treatment, as well as in addressing conflicting information”. Nationwide accessible media such as television and radio could alternatively be used to target a group of older diabetic patients with lower income and educational level.

The barriers to information seeking identified in the present study, (basically lack of time, cost, large volume of unorganized information, lack of infrastructures in the rural areas) are similar to the ones reported in the literature [22] and call for the designing of active and personalized information delivery mechanisms.

### Potential limitations

The present study was undertaken in a major University-affiliated Diabetes Center in Greece and thus the participants, the majority of whom resided in an urban area, might not be representative of the general diabetic population in other parts of the country. This is substantiated by their satisfactory diabetes control (HbA1c 6.5-7.5% in 44.8% of them). Those who refused to participate (due to alleged language or visual problems or lack of time) might represent a specific group of people with no interest in information and knowledge about the disease and thus may have introduced a bias in the study.

## Conclusion

In conclusion, the present study investigated reported information needs, resources and obstacles of a cohort of diabetic patients, followed-up in a Diabetes outpatient Clinic in Greece. The presented results may have significant implications in current practice and public health policy regarding DM information behavior in Greece, ultimately leading to further ameliorate the clinical outcomes in a cost-effective way. The physicians are perceived to be of highest importance to the diabetic patients studied. The physicians, if given the possibility, could therefore invest more time in informing patients about DM, especially about the diet and diabetic complications which are reported to be of keen interest to the patients. The value of broadcast media should be understood and the possibility of an organized DM information campaign in television and radio should be evaluated. The disparities in patients’ stated importance between the physicians and other health care providers is of concern. Further involving health care providers other than doctors could represent a significant opportunity to provide better information for diabetic patients (i.e. dieticians, diabetes educators). Internet on the other hand could be used to target younger patients and has the potential to be an important information resource in the future, as diabetic patients become progressively more computer literate and experienced in internet use. Further studies are warranted in order to validate the findings of the present study and to elucidate the information behavior of diabetic patients, ideally testing the value of a multimodality information intervention strategy in terms of clinical outcomes and cost effectiveness. Further research is also required to determine if the perception of satisfaction with information that the participants expressed is equal to actual knowledge about these issues when tested. The potential should be investigated in future studies, not only whether satisfaction with information and education is associated with a correct understanding of diabetes-related issues, but also whether this knowledge is translated into healthy behaviors.
